# Residency Program Director Perspectives on Changes to US Medical Licensing Examination

**DOI:** 10.1001/jamanetworkopen.2021.29557

**Published:** 2021-10-15

**Authors:** Andrew Wang, Krystal L. Karunungan, Jacob D. Story, Edward L. Ha, Clarence H. Braddock

**Affiliations:** 1 College of Medicine, Charles R. Drew University of Medicine and Science, Los Angeles, California; 2 David Geffen School of Medicine, University of California, Los Angeles

## Abstract

This survey study examines the perspectives of residency program directors regarding reporting the US Medical Licensing Examination (USMLE) Step 1 as pass/fail and discontinuing Step 2 Clinical Skills.

## Introduction

In recent landmark decisions on February 12, 2020, and January 26, 2021, the United States Medical Licensing Examination (USMLE) cosponsors announced the adoption of reporting Step 1 pass/fail and the discontinuation of Step 2 Clinical Skills (CS), respectively. These changes were met with mixed reviews from program directors and medical students applying to residency.^1,2^ In the National Resident Matching Program’s (NRMP) 2018 survey, 78% of program directors (PD) reported that they cite Step 1/Comprehensive Osteopathic Medical Licensing Examination (COMLEX) Level 1 when reviewing applications, compared with 70% of PDs for Step 2 Clinical Knowledge (CK)/COMLEX 2 Performance Evaluation (PE) and 51% for Step 2 CS/COMLEX 2 Cognitive Evaluation (CE). Conversely, Step 2 CS was rated as slightly more important than Step 1 and Step 2 CK, supporting the discontinued examination’s value when ranking applicants. However, with Step 1 now reported as pass/fail and Step 2 CS discontinued, there remains uncertainty regarding how PDs will tailor their review of applications. Understanding PDs’ perspectives on these consequential changes can guide educators reshaping their curricula and students aiming to strengthen their candidacy for residency.

## Methods

The authors (A.W., J.D.S., K.L.K.) manually queried a subset (1600 of more than 5000, outreach >50% for every medical specialty except internal medicine and family medicine) of valid PD emails through the Accreditation Council for Graduate Medical Education’s public 2019 to 2020 List of Specialty Programs (n = 31) across all medical specialties. In rounds, PDs were allotted 3 months (January to April 2021) to respond to the survey, with a reminder email sent after the first week. The University of California at Los Angeles institutional review board deemed this study exempt from review and waived informed consent because it used deidentified data. This study followed the American Association for Public Opinion Research (AAPOR) reporting guideline.

We created a 14-item anonymous online survey using the ExpertReview validation tool (Qualtrics XM operating system version X4 [Qualtrics International Inc]) (eTable 1 in the Supplement). The survey (using Qualtrics and Google Forms) included questions on PD demographics including age, gender, tenure, and residency program specialty. PD race and ethnicity data were not collected to preserve anonymity. PDs were prompted for their general perceptions regarding the impact of residency selection in the context of changes to USMLE Step 1 and Step 2 CS. Responses were recorded as binary (yes or no) or on 3-point Likert scales (disagree, neutral, or agree) or 5-point Likert scales (strongly disagree, disagree, neutral, agree, or strongly agree).

Categorical variables were reported as counts and percentages. Derived 95% CIs were from the margin of errors of total sample (±3.1%) and subgroups (±4.3%) defined by AAPOR (eTable 1 in the Supplement). Subgroup analyses between regions and between Association of American Medical Colleges (AAMC)–defined primary care (internal medicine, family medicine, pediatrics, internal medicine/pediatrics) and nonprimary care specialties were conducted for all results. For final analysis, surveys with incomplete PD demographics were excluded (n = 25) and further grouping analyses were performed by converting 5-point Likert responses to 3-point (strongly disagree and disagree vs neutral vs agree and strongly agree). Incomplete PD surveys (<3%) were censored for each response. Statistically significance (*P* < .05) was considered with plurality of Likert 3-point responses by nonoverlapping 95% CI. Statistical analysis was performed using Stata statistical software version 16.1 (StataCorp) from February to June 2021. 

## Results

The total survey response rate was 55.7% (n = 891), with 97.2% of responses (n = 866) included in the final synthesis. Participants were more commonly male (62.7% [n = 543]) and broadly represented across the 4 US regions (Northeast: 29.3% [n = 252]; South: 28.6% [n = 248]; Midwest: 23.0% [n = 199]; West: 19.1% [n = 167]). The highest response rates were from family medicine (66.5% [n = 133]), pediatrics (64.3% [n = 45]) and orthopedic surgery (64.0% [n = 32]). In total, family medicine (15.4% [n = 133]), internal medicine (10.1% [n = 88]), and surgery (6.6% [n = 57]) were the most commonly represented specialties (Table 1). More responses from nonprimary care specialties (65.2% [n = 565]) were collected than primary care (34.8% [n = 301]).

**Table 1.** Demographic Characteristics of Residency Program Director Survey Respondents by Specialty

Specialty	Response rate
	Respondents, No./total No. (%)
Allergy and immunology	25/80 (31.3)
Anesthesiology	21/40 (52.5)
Child neurology	11/20 (55.0)
Colon and rectal surgery	17/60 (28.3)
Dermatology	23/40 (57.5)
Emergency medicine	48/90 (53.3)
Family medicine	133/200 (66.5)
Internal medicine	88/150 (58.7)
Internal medicine/pediatrics	35/60 (58.3)
Interventional radiology (integrated and independent)	11/20 (55.0)
Medical genetics and genomics	5/10 (50.0)
Neurologic surgery	19/30 (63.3)
Neurology	25/40 (62.5)
Nuclear medicine	5/10 (50.0)
Obstetrics and gynecology	55/100 (55.0)
Ophthalmology	22/40 (55.0)
Orthopedic surgery	32/50 (64.0)
Otolaryngology	17/30 (56.7)
Pathology	16/30 (53.3)
Pediatrics	45/70 (64.3)
Physical medicine and rehabilitation	9/20 (45.0)
Plastic surgery (integrated and independent)	17/30 (56.7)
Preventive medicine	10/20 (50.0)
Psychiatry	44/80 (55.0)
Radiation oncology	15/30 (50.0)
Radiology-diagnostic	24/50 (48.0)
Surgery	57/90 (63.3)
Thoracic surgery	11/20 (55.0)
Transitional year	16/30 (53.3)
Urology	24/40 (60.0)
Vascular surgery (integrated)	11/20 (55.0)

From Table 2, many PDs reported that they will place greater emphasis on tiered core clerkship grades (87.7%; 95% CI, 84.6%-90.8%; n = 759), Step 2 CK (83.4%; 95% CI, 80.1%-86.5%; n = 722) and NBME subject examinations (40.5%; 95% CI, 37.4%-43.6%; n = 351). In addition, the majority of PDs reported that clerkship narrative assessments (75.2%; 95% CI, 72.1%-78.3%; n = 651), subinternship and away evaluations (74.2%; 95% CI, 71.1%-77.3%; n = 643), and reference letters (67.6%; 95% CI, 64.5%-70.7%; n = 585) will become more important. When stratifying by specialty, 72.9% (95% CI, 68.6%-77.2%, n = 469) of PDs from nonprimary care specialties will increase emphasis on reference letters compared with 23.5% (95% CI, 27.8%-27.8%, n = 52) from primary care.

**Table 2.** Program Directors’ Perspectives on Which Application Elements They Will Emphasize Following the Discontinuation of Step 2 Clinical Skills and Pass/Fail Scoring of Step 1

Residency applicant selection factors	Responses, No.	% (95% CI)				
		Strongly disagree	Disagree	Neutral	Agree	Strongly agree
Overall core clerkship grades	863	3.3 (0.2-6.4)	1.7 (0.0-4.8)	7.3 (4.2-10.4)	40.4 (37.3-43.5)	47.3 (44.2-50.4)
Clerkship narrative assessment	857	5.0 (1.9-8.1)	4.9 (1.8-8.0)	14.9 (11.8-18.0)	41.5 (38.4-44.6)	33.7 (30.6-36.8)
Medical school prestige	860	6.9 (3.8-10.0)	18.8 (15.7-21.9)	28.1 (25.0-31.2)	26.8 (23.7-29.9)	19.4 (16.3-22.5)
NBME scores	857	8.6 (5.5-11.7)	18.1 (15.0-21.2)	32.8 (29.7-35.9)	19.4 (16.3-22.5)	21.1 (18.0-24.2)
Step 2 CK	860	0.4 (0.0-3.5)	3.6 (0.5-6.7)	12.6 (9.5-15.7)	25.6 (22.5-28.7)	57.8 (54.7-60.9)
Reference letters	863	7.7 (4.6-10.8)	9.9 (6.8-14.0)	14.8 (11.7-17.9)	31.1 (28.0-34.2)	36.5 (33.4-39.6)
Subinternship evaluation	857	3.2 (0.1-6.3)	4.2 (1.1-7.3)	18.4 (15.3-21.5)	36.8 (33.7-39.9)	37.4 (34.3-40.5)
Personal statement	854	5.4 (2.3-8.5)	14.2 (11.1-17.3)	36.2 (33.1-39.3)	37.6 (34.5-40.7)	6.6 (3.5-9.7)
Research experience	860	6.6 (3.5-9.7)	10.4 (7.3-13.5)	41.0 (37.9-44.1)	35.3 (32.2-38.4)	6.7 (3.6-9.8)
Community service	860	5.2 (2.1-8.3)	13.7 (10.6-16.8)	38.1 (35.0-41.2)	37.3 (34.2-40.4)	5.7 (2.6-8.8)
Leadership experience	863	2.6 (0.0-5.7)	8.6 (5.5-11.7)	33.3 (30.2-36.4)	45.4 (42.3-48.5)	10.1 (7.0-13.2)
Academic awards or special honor societies	860	3.1 (0.0-6.2)	4.5 (1.4-7.6)	25.4 (22.3-28.5)	37.7 (34.6-40.8)	29.3 (26.2-32..4)

Abbreviations: CK, Clinical Knowledge; NBME, National Board of Medical Examiners.

Across specialties, many PDs will consider medical school prestige (46.2%; 95% CI, 43.1-49.3, n = 400), leadership experience (55.5%; 95% CI, 52.4-58.6, n = 481), and academic awards or honor society membership (67.0%; 95% CI, 63.9-70.1, n = 580) as more important. Although there was no consensus on whether PDs will place greater emphasis on the personal statement, community service, or research, PDs from nonprimary care specialties did report that the personal statement (42.8%; 95% CI, 38.5%-47.1%, n = 275) and research (72.9%; 95% CI, 68.6%-77.2%, n = 469) will become more important.

## Discussion

Our findings suggest that PDs will consider evaluations of clinical core competency as even more important moving forward. However, narrative assessments and subinternship evaluations may be limited in their utility given increasing concern regarding racial, ethnic and gender disparities in grading,[3] further highlighting the increasing need for standardized measures of clinical performance.

Reduced competition and improved student wellness are commonly cited benefits of pass/fail scoring.^4^ However, the vast majority of PDs responded that they would increase emphasis on Step 2 CK and NBME subject examinations, suggesting that PDs remain resolute in using standardized scores when reviewing applications. Without Step 2 CS and numerical scores for Step 1, heightened academic pressure may be placed on students to perform well on Step 2 CK as the only standardized metric.

With the quantitative changes to USMLE, PDs may be encouraged to consider other application components. Although only PDs from nonprimary care specialties reported that the personal statement will become more important, PDs across specialties will consider leadership experience and academic awards or honor society membership as more important. The latter finding is particularly important as rising concern regarding racial disparities in the Alpha Omega Alpha (AΩA) Honor Medical Society membership has led medical schools to reevaluate their AΩA affiliations.^5^ Lastly, PDs from nonprimary care specialties reported that research experience will become more important, supporting existing NRMP trends of nonprimary care specialties requiring greater research involvement.

Although these findings support PDs reviewing nonnumerical components of the application, some PDs reported that they will increase emphasis on medical school prestige. Standardized tests provide an opportunity for graduates from international or less prestigious schools to demonstrate achievement, and these groups may be disadvantaged by the removal of objective scores. Furthermore, prior studies have suggested that PDs may attribute substantial weight to the status and rank of the authors of reference letters,^6^ and students at prestigious schools may be more likely to obtain letters from recognizable faculty. In our sample, the majority of PDs reported that reference letters will become more important with PDs from nonprimary care specialties showing a greater increase.

Several limitations of this brief study should be considered. First, because PDs were asked to consider how they would change their review of residency applications immediately after the changes to USMLE, availability heuristic bias may have occurred as PDs may not have had adequate time to formulate their responses. Second, although the total number of respondents was high, the overall response rate across all specialties was insufficient to avoid selection bias or allow for subspecialty analysis which limits generalizability. This study included a broad representation of specialties with the vast majority of uncollected emails being internal and family medicine. However, no difference was observed during subgroup (primary vs nonprimary, regional variation) and sensitivity analysis.

PDs favor using numerical scores to differentiate applicants.^5^ With Step 2 CK becoming the sole standardized metric provided to residency programs, our findings suggest that greater importance will be placed in an applicant’s core clerkship performance, leadership experience, academic awards and honor society memberships, and reference letters. Indeed, additional work is needed to develop a residency application process that satisfies the necessity for objective metrics while sufficiently recognizing nonacademic achievements, and this can only be accomplished through active communication among stakeholders including medical students, educators, and residency PDs.

## Notes

Supplement. **eTable.** AAPOR Disclosure Checklist
