# Supplementary material for: Residency Program Director Perspectives on Changes to US Medical Licensing Examination
Source: JAMA Netw Open. 2021 Oct 15;4(10):e2129557. doi: 10.1001/jamanetworkopen.2021.29557 (PMC8520127; doi:10.1001/jamanetworkopen.2021.29557)
Supplement: Supplement. — eTable. AAPOR Disclosure Checklist [file jamanetwopen-e2129557-s001.pdf]

Supplemental Online Content

Wang A, Karunungan KL, Story JD, Ha EL, Braddock CH. Residency program director perspectives on changes to US Medical Licensing Examination. *JAMA Netw Open.* 2021;4(10):e2129557. doi:10.1001/jamanetworkopen.2021.29557

eTable. AAPOR Disclosure Checklist

This supplemental material has been provided by the authors to give readers additional information about their work.

© 2021 Wang A et al. *JAMA Network Open.*

eTable. AAPOR Disclosure Checklist

BASIC DISCLOSURE ELEMENTS	DETAILS
Survey sponsor	UCLA
Survey/Data collection supplier	Qualtrics/Google Form
Population represented	Program Directors listed ACGME Specialty List 2019-2020
Sample size	1600 Valid Emails
Mode of data collection	Anonymous Online Survey
Type of sample	Probability, for individual emails. Queried outreach >50%+ for every medical specialty except internal medicine and family medicine.
Start and end dates of data collection	January 2021 to April 2021
Margin of sampling error for total sample	+/- 3.1 percentage points at the 95% confidence level
Margin of sampling error for key subgroups	+/- 4.3 percentage points at the 95% confidence level
Are the data weighted?	No, however, primary care vs non primary care and regional differences subgroup analysis were performed
Is the data censored?	Yes, to individual response question
Contact for more information	Andrew Wang<br>David Geffen School of Medicine<br>andrewwang@mednet.ucla.edu

© 2021 Wang A et al. *JAMA Network Open.*
